# Infectious keratitis secondary to canaliculitis with concretions: A case report

**DOI:** 10.1097/MD.0000000000017444

**Published:** 2019-10-04

**Authors:** Yu-Pu Chou, Po-Han Yeh, Yueh-Ju Tsai, Chieh-Hung Yen, Ching-Hsi Hsiao

**Affiliations:** a Department of Education, Chang Gung Memorial Hospital, Linkou; b Department of Medicine, Mackay Medical College, New Taipei City; c Department of Ophthalmology, Chang Gung Memorial Hospital, Linkou; d College of Medicine, Chang Gung University, Taoyuan, Taiwan.

**Keywords:** canalicular concretions, canaliculitis, infectious keratitis

## Abstract

**Rationale:**

Canaliculitis is a frequently overlooked and misdiagnosed disease. Concurrent corneal ulceration with canaliculitis is uncommon. We report such a case.

**Patient concerns:**

An 87-year-old woman complained of swelling and pain of the right eye after acute angle closure glaucoma attack. Slit-lamp examination was compatible with the features of infectious keratitis, and the cultures from corneal scrapings grew *Streptococcus anginosus* later. Hourly topical vancomycin (25 mg/ml) was instilled, then the corneal ulceration improved initially but became stationary after 1-week treatment.

**Diagnosis:**

Discharge from the upper punctum was noted subsequently and canalicular concretions were found through curettage. The cultures from canalicular discharge and concretions also revealed the presence of *S. anginosus*. Thus, infectious keratitis secondary to canaliculitis was diagnosed.

**Interventions:**

Canaliculotomy was performed to remove the large concretion and vancomycin was injected locally.

**Outcomes:**

The corneal ulceration resolved after canaliculitis was appropriately treated.

**Lessons:**

Canaliculitis could be a reservoir for organisms that may make compromised corneas liable to infections. Only the appropriate diagnosis and aggressive treatment of canaliculitis leads to the eradication of associated corneal infections.

## 1 Introduction

Canaliculitis, an infection of the canalicular part of the lacrimal system, is frequently misdiagnosed, despite its well-documented clinical presentations, such as punctal or canalicular swelling, expressible punctal discharge, and a pouting punctum.^[1]^*Actinomyces* species were previously the commonest causative organisms of lacrimal canaliculitis and canalicular concretions, which are common findings in patients with canaliculitis;^[1]^ however, recent studies have revealed a higher prevalence of streptococcal and staphylococcal infections.^[1–3]^ Medical treatment is rarely effective in clearing canaliculitis, partly because canalicular concretions serve as reservoirs for bacteria and protect bacteria from lethal antibiotic concentrations.^[1]^ Surgical management, such as dilation and curettage or canaliculotomy, represents the definitive therapy for canaliculitis.^[1–3]^

Infectious keratitis can cause corneal perforation and scarring and can result in serious visual impairment. It continues to be a predominant part of ocular morbidity throughout the world. Accordingly, infectious keratitis must be treated instantly, predominantly by topical antibiotics, but when medical treatment fails, surgical intervention is required. Various predisposing factors for infectious keratitis, such as ocular trauma, the wearing of contact lenses, ocular surface disorder, ocular surgery, systemic diseases, and topical corticosteroid usage have been reported.^[4,5]^ However, the link between infectious keratitis and neighboring tissues has not been thoroughly explored. Herein, we report the case of a patient with infectious keratitis secondary to canaliculitis with concretions. The infection was finally controlled after the surgical removal of canalicular concretions.

## 2 Case presentation

**Figure 1:**
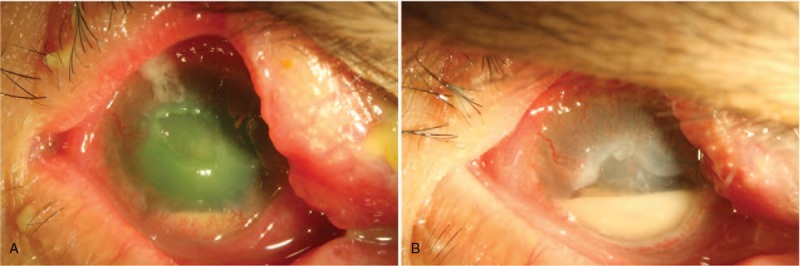
Slit-lamp photographs of the patient. A. A corneal epithelial defect with infiltrate, accompanied by a 2-mm hypopyon. Superficial corneal neovascularization was covered over superior and inferior peripheral cornea. Viewed retrospectively, swollen and pouted upper punctum was noted. B. Deep ulcerated cornea without prominent infiltrate, accompanied by a 4-mm hypopyon after 7-day instillation of topical vancomycin.

An 89-year-old woman complained of swelling and pain of the right eye for 10 days. She was diagnosed with acute angle closure glaucoma in the right eye and was treated through the intravenous injection of mannitol, oral acetazolamide, topical carteolol, brimonidine, and fluorometholone at another hospital. She had received trichiasis correction in the right eye several years previously. Apart from hypertension, the patient did not have any systemic diseases. Upon examination, her visual acuity was light perception and intraocular pressure was 32 mm Hg in the right eye. Slit-lamp examination revealed a 4 × 3 mm corneal epithelial defect with infiltrate accompanied by a 2-mm hypopyon, and superficial corneal neovascularization was covered over superior and inferior peripheral cornea (Fig. 1A), and mature cataract in the right eye. Corneal scrapings were sent for cultures, including for bacteria, mycobacteria, and fungi. The patient was initially administered hourly topical levofloxacin; fluorometholone was discontinued. The corneal culture grew *Streptococcus anginosus,* which was susceptible to ampicillin, ceftriaxone, teicoplanin, and vancomycin, but resistant to erythromycin and clindamycin. On the follow-up visit, it was revealed that the right cornea continued melting and hypopyon increased to 4 mm height; therefore, she was admitted as an inpatient and placed on hourly topical vancomycin (25 mg/ml) instead. After the 7-day instillation of topical vancomycin, the cornea cleared but hypopyon was stationary (Fig. 1B); thus, 1% betamethasone 4 times per day was administered to determine if inflammation played a role in her condition. However, the patient's condition deteriorated slightly, so corneal culture was performed again to rule out polymicrobial infection or the emergence of resistant strains. The following day, a substantial amount of discharge from the upper punctum was noted. Lacrimal syringing was patent, and canalicular concretions were found through curettage; canaliculotomy was performed to remove the large concretion and vancomycin was injected locally. Cultures of corneal scrapings and canicular discharge/concretions also revealed the presence of *S. anginosus*. Subsequently, amniotic membrane transplantation was performed to promote reepithelialization. The corneal ulcer healed gradually and the patient was discharged and prescribed topical vancomycin (25 mg/ml) and betamethasone 1% 4 times a day and brimonidine solution twice a day. At 1-year follow-up, visual acuity of the right eye was restricted to hand motions due to central corneal scarring and mature cataract.

This study was approved by the Institutional Research Ethics Board at Chang Gung Memorial Hospital, Taiwan.

## 3 Discussion

We report a case of infectious keratitis with unnoticed canaliculitis. Both corneal and canalicular discharge/concretions grew the same organism (*S. anginosus*) with the same antibiotic susceptibility. The corneal infection did not resolve completely after the administration of topical antibiotics until the canaliculitis and concretions were appropriately treated. It is likely that infectious keratitis was secondary to canaliculitis in this patient.

Concurrent corneal ulceration with canaliculitis is uncommon. In a previous 10-year (from 2003 to 2012) study of microbial keratitis in our hospital,^[5]^ none of the cases of patients having microbial keratitis were related to canaliculitis. At the time of writing, 4 cases of concurrent corneal ulceration and canaliculitis have been reported in the literature (Table 1). Feder et al reported a patient with a Smart-PLUG installed who had *Mycobacteria chelonae* keratitis and canaliculitis.^[6]^ Although the patient responded favorably to topical therapy, *M. chelonae* was still present in the host cornea following penetrating keratoplasty. Yokogawa et al reported a case series of patients who underwent surgical therapy for corneal perforations;^[7]^ corneal perforations in 2 of the 31 patients were caused by canaliculitis. Although the concretions of these 2 patients grew anaerobic bacteria, these perforations were classified as noninfectious, and it was speculated that they were caused by allergies against toxins produced by bacteria.^[7]^ Both patients were cured by surgical removal of canalicular concretions, and lamellar keratoplasty and amniotic membrane transplantation for corneal perforations.^[7]^ Recently, Ishikawa and Kato reported the case of a patient with ocular cicatricial pemphigoid who developed corneal perforation, which was presumably caused by the adherence of bacterial concretions from lacrimal canaliculitis.^[8]^ Punctoplasty and the removal of canalicular concretions were performed, and histopathology revealed sulfur granules produced by *Actinomyces* spp.^[8]^ Combined these 4 patients with our case, there are certain characteristics of concurrent corneal ulceration and canaliculitis (Table 1). First, other risk factors for corneal ulcer, such as dry eye disease^[6]^ and ocular cicatricial pemphigoid,^[8]^ were found. Our patient might have had an ocular surface disease based on the presence of corneal neovascularization and the history of trichiasis operation, or an epithelial corneal defect caused by bullae formation secondary to high intraocular pressure. Second, cultures from canalicular discharge and/or concretions were positive in all patients. Furthermore, the patient in the case report of Feder et al^[6]^ and our patient had the same organism cultured from both corneal scrapings and canicular discharge. Third, except for patients with secondary canaliculitis caused by the implantation of a Smart-PLUG,^[6]^ those patients with primary canaliculitis underwent surgical removal of concretions to cure the infection. Thus, we deduced that the possible pathogenesis of concurrent corneal ulceration and canaliculitis in these patients was canaliculitis/concretions converting canaliculi, and even conjunctival sac, into a reservoir of organisms, resulting in compromised corneas liable to infections. To cure such a corneal infection, the accurate diagnosis and aggressive treatment of canaliculitis are first necessary.

**Table 1:**
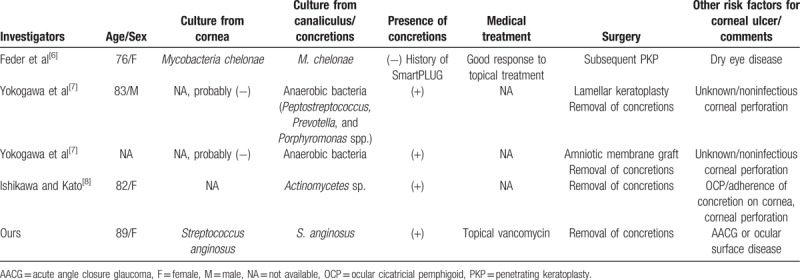
Clinical data of the patients with corneal ulceration and canaliculitis.

## 4 Conclusion

In conclusion, this case highlights the importance of assessing the neighboring tissues in patients with infectious keratitis, particular when the corneal infection could not be eradicated with appropriate antibiotics. Canaliculitis, a frequently overlooked and misdiagnosed disease, could be a source of infection in compromised corneas. Early and appropriate diagnosis and surgical treatment, such as dilatation, curettage, or canaliculotomy to remove all concretions, can eradicate canaliculitis and keratitits, preventing further complications.

## Author contributions

**Conceptualization:** Ching-Hsi Hsiao.

**Data curation:** Po-Han Yeh.

**Investigation:** Po-Han Yeh, Yueh-Ju Tsai, Chieh-Hung Yen, Ching-Hsi Hsiao.

**Supervision:** Yueh-Ju Tsai, Ching-Hsi Hsiao.

**Writing – original draft:** Yu-Pu Chou.

**Writing – review & editing:** Ching-Hsi Hsiao.
